# Not always protective: health literacy, school connectedness and adolescent healthy eating behaviors—the moderating role of physical activity participation

**DOI:** 10.3389/fnut.2026.1976575

**Published:** 2026-10-05

**Authors:** Jiawei Chen, Mengfen Liu, Xinjie Han, Yanxia Tang

**Affiliations:** 1 College of Physical Education and Sports Science, Jishou University, Jishou, China; 2 Department of General Education, Hunan Mechanical & Electrical Polytechnic, Changsha, Hunan, China; 3 Department of Biomechanics and Sport Engineering, Gdansk University of Physical Education and Sport, Gdańsk, Poland; 4 Normal College, Jishou University, Jishou, China

**Keywords:** a moderated mediation model, eating behaviors, health literacy, physical activity participation, school connectedness

## Abstract

**Purpose:**

To explore the effects of Health literacy on adolescent healthy eating behaviors, a moderated-mediation model was constructed in this study to examine the mediating effect of School connectedness and the moderating effect of Physical activity participation.

**Methods:**

Stratified cluster sampling was adopted. Questionnaires were administered to 3, 009 middle-school students recruited from 12 middle schools across 6 provinces nationwide, using the Health literacy scale, Eating behaviors scale, School connectedness scale, and Physical activity participation scale. Data analyses were performed using SPSS 26.0 and PROCESS Model 15.

**Results:**

(1) Health literacy was significantly and positively associated with adolescent healthy eating behaviors. (2) A statistically significant indirect effect through School connectedness was identified; however, its direction was opposite to the hypothesized positive mediation, indicating a suppression pattern. (3) Physical activity participation significantly moderated the direct association between Health literacy and healthy eating behaviors, with this positive association progressively weakening as Physical activity participation increased. (4) Physical activity participation also moderated the second stage of the indirect pathway (School connectedness → healthy eating behaviors). The suppression pattern was stronger at low levels of Physical activity participation but was attenuated and statistically reversed at high levels, where the conditional indirect effect became weakly positive.

**Conclusion:**

Adolescent healthy eating behaviors are shaped by the interactive effects of individual cognition, school environments, and adolescents’ motivational tendency for sports-participation within institutional school physical-activity contexts. School connectedness may function as a counteracting factor in the association between Health literacy and healthy Eating behaviors; however, this counteracting effect is contingent upon Physical activity participation, disappearing and shifting toward a weak positive effect at high levels of Physical activity participation. These findings highlight the importance of jointly considering Health literacy, School connectedness, and Physical activity participation in school-based health promotion. However, given the cross-sectional design and the small positive indirect effect observed at high levels of Physical activity participation, further longitudinal and intervention studies are needed before specific stratified intervention strategies are recommended.

## 1 Introduction

Poor dietary patterns and insufficient physical activity constitute a major global public health burden, particularly among adolescent populations (1, 2). Over the past three decades, the consumption of ultra-processed foods has increased dramatically. The co-occurrence of unhealthy diets and sedentary lifestyles significantly elevates the incidence risk of obesity, metabolic disorders, and emotional and psychological disturbances among adolescents (3, 4). School connectedness reflects adolescents’ school belonging, peer bonding, and school engagement, and serves as a critical determinant of individual health perceptions (5, 6). In contrast, Health literacy governs adolescents’ ability to recognize health information and comprehend nutritional knowledge, which can be further translated into stable Eating behaviors (7, 8). Against this global public health backdrop, this study clarifies the mediating mechanism through which Health literacy shapes adolescent eating behaviors and identifies Physical activity participation as a critical boundary condition. The findings provide localized empirical evidence for the implementation of integrated school-based intervention programs combining nutritional health and physical education practices.

Existing studies have demonstrated that complex intrinsic associations exist among Health literacy, School connectedness, Eating behaviors, and Physical activity participation (9, 10). Higher levels of Health literacy enable individuals to acquire dietary and nutritional knowledge, develop scientific dietary perceptions, and ultimately foster healthy Eating behaviors (11). Meanwhile, the level of adolescent school adaptation and School connectedness is significantly shaped by Health literacy. Adolescents with superior health cognition are more willing to participate in collective school activities, thereby attaining enhanced school belonging (12, 13). As a critical school-based psychosocial resource, school connectedness positively facilitates individual engagement in physical activities and further exerts predictive effects on daily dietary choices (14, 15). As a pivotal motivational-oriented lifestyle-related variable among Chinese secondary school students, Physical activity participation reflects adolescents’ sports-participation motivation, which shapes their subjective investment within institutional school physical-activity contexts and modulates the magnitude of the translational effect from individual psychosocial resources to eating behaviors. Although the associations among the above variables have been validated in prior studies, the existing literature presents notable limitations. Most previous research has focused merely on simple bivariate correlations, failing to explain the sequential mechanism linking Eating behaviors to changes in school belonging. Furthermore, the moderating role of Physical activity participation has been largely overlooked in existing models. Accordingly, this study takes Eating behaviors as the core outcome variable, incorporates school connectedness as the mediator and Physical activity participation as the moderator, and tests a moderated-mediation model to elucidate the intrinsic mechanism underlying the influence of Health literacy on eating behaviors. The findings provide empirical implications for the promotion of healthy behaviors among adolescents.

### 1.1 The relationship between health literacy and eating behaviors

Health literacy represents a comprehensive capacity for individuals to obtain, comprehend, screen, and process health-related information, as well as to make health-promoting behavioral decisions based on such information (16). In the present study, it specifically refers to adolescents’ cognitive capacity to recognize nutritional knowledge and interpret diet-related information. Eating behaviors describe individual daily dietary choices, food-intake patterns, and dietary habits (17). In this study, it primarily denotes adolescent healthy Eating behaviors, encompassing a set of diet-related behavioral manifestations such as fruit-and-vegetable consumption, maintenance of balanced diets, and reduced intake of high-sugar, high-fat processed foods. It is important to distinguish general Health literacy from more domain-specific constructs related to dietary behaviors, such as nutrition literacy and food literacy. General Health literacy broadly refers to individuals’ capacities to access, understand, evaluate, and apply health-related information across multiple health domains (18). In contrast, nutrition literacy focuses more specifically on the ability to obtain, comprehend, evaluate, and use nutrition-related information to support appropriate dietary decisions, whereas food literacy represents a broader set of knowledge, skills, and practices involved in planning, selecting, preparing, and consuming food. Although these constructs are conceptually related, they are not interchangeable. The present study focuses on general Health literacy because its theoretical interest lies in whether broad health-cognitive resources are associated with adolescents’ healthy Eating behaviors and how this association operates through School connectedness and varies according to Physical activity participation. Nutrition literacy and food literacy may represent more proximal determinants of dietary behavior and therefore warrant separate examination in future studies. Health literacy serves as a critical cognitive antecedent variable that drives individuals’ healthy Eating behaviors (19). Only when individuals possess adequate Health literacy can they accurately identify diet-related health information, distinguish the merits and drawbacks of healthy foods versus ultra-processed foods, and subsequently translate nutritional cognition into practical dietary choices. Existing studies have indicated (20, 21) that individuals with higher Health literacy are more likely to maintain balanced diets, limit the consumption of high-sugar and high-fat foods, and develop healthier Eating behaviors. Conversely, adolescents with insufficient Health literacy struggle to screen misleading dietary information disseminated online and by peers, and are prone to unhealthy Eating behaviors such as food selectivity and frequent consumption of ultra-processed foods. Both large-scale macro-level surveys in public health and empirical evidence from subgroup analyses within school-based settings have confirmed that Health literacy and Eating behaviors are not mutually isolated independent variables, and a distinct direct effect path exists between the two constructs. Accordingly, the present study proposes Hypothesis H1: Health literacy positively predicts adolescent healthy Eating behaviors.

### 1.2 The mediating role of school connectedness

School connectedness refers to adolescents’ perceptions of teacher-student acceptance, peer affirmation, and an overall sense of belonging within school contexts (22, 23). High levels of School connectedness indicate stronger identification with the school community, greater willingness to participate in class- and club-based collective activities, and greater receptiveness to health-related guidance delivered by schools. By contrast, low School connectedness is characterized by weaker attachment to the school community and reduced engagement in collective school activities. Within the conceptual model proposed in the present study, Health literacy is positioned as an antecedent of School connectedness. Previous studies have found that higher levels of Health literacy are associated with greater School connectedness among adolescents (24, 25). Adolescents with adequate Health literacy may be better able to understand health-related information delivered through school health-education curricula and campus health-promotion activities and may therefore be more receptive to the health concepts and behavioral norms advocated by schools. Moreover, adolescents with higher Health literacy may be more willing to participate in school-based health-related activities. Through interactions in these settings, they may receive greater teacher encouragement and peer affirmation, thereby strengthening their sense of belonging and emotional attachment to the school. These processes provide a theoretical basis for the proposed pathway from Health literacy to School connectedness. School connectedness may, in turn, be associated with adolescents’ Eating behaviors (26). Adolescents who perceive greater teacher care, peer support, and school belonging may be more receptive to health-promoting norms communicated within the school environment and may therefore be more likely to adopt positive Eating behaviors, such as maintaining a balanced diet and regular main-meal consumption, while reducing maladaptive Eating behaviors such as binge eating and excessive consumption of snacks and sugary beverages. Consistent with this perspective, previous studies have indicated that a positive school climate is associated with fewer risky health behaviors and healthier lifestyle patterns among adolescents (27). These findings provide a theoretical basis for positioning School connectedness as a mediator linking Health literacy to Eating behaviors. Nevertheless, the relationship between School connectedness and Eating behaviors may not be strictly unidirectional. Existing school-health research also suggests that adolescents’ dietary patterns may influence their school-based social experiences. Regular and balanced dietary habits may facilitate peer dining and other social interactions, whereas unhealthy dietary patterns may be associated with less favorable social experiences and weaker perceptions of School connectedness (28). Accordingly, reciprocal relationships between School connectedness and Eating behaviors are theoretically possible. The directional ordering adopted in the present study therefore reflects the theoretically specified pathway of Health literacy → School connectedness → Eating behaviors rather than an assumption of established temporal causality. Given the cross-sectional design, the present data cannot determine the temporal ordering of School connectedness and Eating behaviors or exclude possible reciprocal relationships. Although the preceding theoretical and empirical evidence primarily supports a positive mediating pathway through School connectedness, School connectedness may not invariably operate in a health-promoting direction. Higher School connectedness may involve deeper immersion in campus peer networks, and peer subcultures within schools are heterogeneous. In addition to school-sanctioned healthy lifestyle norms, peer groups may also transmit maladaptive dietary practices, such as frequent snack sharing, take-out consumption, and collective overeating (29, 30). Adolescents with a strong sense of school belonging may conform to such peer dietary norms to maintain social acceptance. Under these circumstances, the indirect association between Health literacy and healthy Eating behaviors through School connectedness could potentially operate in a direction opposite to the positive direct association. Taken together, the primary theoretical expectation is that higher Health literacy is associated with greater School connectedness, which, in turn, is associated with healthier Eating behaviors. Accordingly, the following directional hypothesis is proposed: H2: School connectedness positively mediates the association between Health literacy and adolescents’ healthy Eating behaviors. An indirect effect operating in the opposite direction is treated as an exploratory finding rather than as evidence supporting H2.

### 1.3 The moderating role of physical activity participation

Physical activity participation refers to behavioral engagement in diverse physical-activity and exercise-related activities, including physical-education classes, extracurricular sports activities, and self-initiated exercise. It provides the motivational foundation for individuals’ practical health-related behaviors and serves as a critical psychological protective factor for fostering healthy lifestyles among adolescents (31, 32). For Chinese secondary school students who are provided with mandatory school physical-education arrangements, higher motivation usually predicts more active subjective engagement in existing physical-activity scenarios. Health literacy represents a cognition-based health resource at the individual level, and its effects on Eating behaviors are constrained by adolescents’ levels of Physical activity participation. Prior studies generally hold that high Physical activity participation amplifies the positive effects of health-related cognition on Eating behaviors (33). Nevertheless, other studies suggest that when adolescents devote substantial energy to physical exercise, exercise-driven nutritional demands and dietary choices within exercise-related contexts attenuate the direct driving force exerted by Health literacy on everyday Eating behaviors (34, 35). This moderating pattern may be further understood through a behavioral automaticity and habit-formation mechanism. Health literacy primarily represents a cognitive resource that supports the deliberate evaluation and selection of health-related behaviors, whereas repeated engagement in physical activity may provide relatively stable behavioral cues and recurring lifestyle contexts in which exercise-related dietary practices become increasingly routinized. As these exercise–diet routines become more established, everyday dietary decisions may rely relatively less on the deliberate application of Health literacy and more on habitual behavioral patterns embedded within physical-activity contexts. Even among individuals with adequate Health literacy, under conditions of high Physical activity participation, part of the behavioral support for dietary choices may therefore derive from established exercise-related routines and nutritional demands, reducing the unique contribution of Health literacy to Eating behaviors. In contrast, individuals with low Physical activity participation may have fewer routine-based behavioral cues and habitual supports derived from exercise engagement. Their Eating behaviors may therefore rely more heavily on cognitive resources such as Health literacy, resulting in a stronger positive association between Health literacy and Eating behaviors. From this perspective, Physical activity participation may function as a boundary condition that shifts the relative contribution of deliberative health-related cognition and habitual behavioral regulation to Eating behaviors. Accordingly, Hypothesis H3a is proposed: Physical activity participation moderates the direct path between Health literacy and Eating behaviors, such that the positive association between Health literacy and Eating behaviors is expected to become weaker as Physical activity participation increases.

School connectedness represents adolescents’school-based sense of belonging and social support from teachers and peers. Nevertheless, this socio-emotional resource exhibits boundary conditions in its effects on Eating behaviors; it does not automatically translate into healthy Eating behaviors, and its effectiveness depends on the school-activity contexts in which individuals participate. From the social-ecological perspective, social bonds can translate into health-promoting behaviors only when a sense of belonging is aligned with health-oriented collective activities (36).

Empirical evidence from adolescent populations provides support for distinguishing sport-oriented contexts from other peer-interaction settings. In a nationally representative sample of U.S. high-school students, sports participation was associated with several favorable health behaviors, including greater fruit and vegetable consumption among some groups (32). Similarly, a large school-based study of Norwegian adolescents found that participation in organized sports was associated with lower odds of irregular main-meal consumption, high intake of unhealthy foods and beverages, and low intake of healthy foods (37). These findings suggest that organized sport may constitute a distinct social and behavioral context in which adolescents are exposed to health-oriented routines and norms that differ from those operating in other everyday peer settings. When adolescents exhibit high levels of Physical activity participation and frequently engage in physical-education classes and group sports, sports-related contexts may provide greater exposure to school-endorsed norms for healthy lifestyles, enabling students to translate the sense of belonging derived from School connectedness into healthy dietary practices. By contrast, among adolescents with low Physical activity participation, even with high School connectedness, peer interactions may be less embedded in such health-oriented activity contexts and may instead occur more frequently in general social settings, in which dietary norms are potentially more heterogeneous. Thus, School connectedness may not necessarily translate into healthy dietary choices in the same manner across different physical-activity contexts. Accordingly, it is hypothesized that the magnitude and even the direction of the predictive effect of School connectedness on Eating behaviors are moderated by Physical activity participation. Accordingly, Hypothesis H3b is proposed: Physical activity participation moderates the second-stage of the mediating path—namely, the relationship between School connectedness and Eating behaviors.

Taken together, this study constructs a moderated-mediation model to examine the effects of adolescents’Health literacy on Eating behaviors, as well as the mediating role of School connectedness and the moderating role of Physical activity participation (see Figure 1). This model further examines the potential pathways and boundary conditions underlying the association between Health literacy and adolescents’ Eating behaviors.

**Figure 1:**
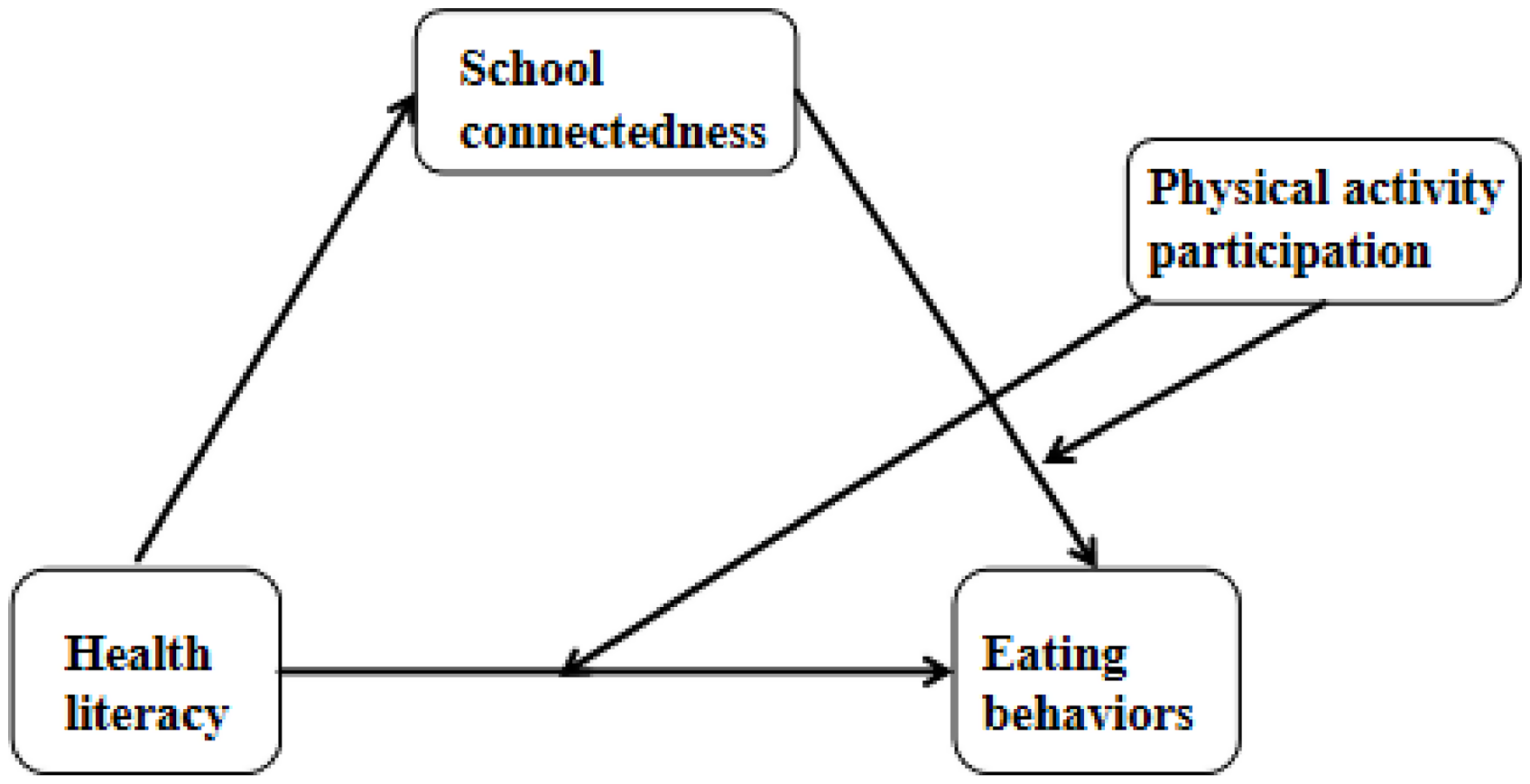
Research framework.

## 2 Methods

### 2.1 Participants and procedures

From March to June 2026, stratified cluster sampling was adopted in this study, and questionnaire-based surveys were conducted via the Wenjuanxing online platform. Participants were middle-school students recruited from 12 secondary schools across six provinces, including Hunan, Hubei, Guangxi, Henan, Hebei, and Yunnan, with ages ranging from 15 to 18 years. The sample consisted of 1730 boys (57.49%) and 1, 279 girls (42.51%). The research protocol was approved by the Ethics Committee of Hunan Mechanical & Electrical Polytechnic (Approval No: 2026051). All survey procedures were performed in strict accordance with the ethical principles outlined in the Declaration of Helsinki. Given that all participants were minors, electronic written informed consent was obtained from their parents or legal guardians prior to survey administration. The informed-consent document clearly stated the research objectives, questionnaire-based data collection procedures, data anonymization protocols, participants’right to withdraw from the study unconditionally at any time without any adverse consequences, and the scope of data utilization. Homeroom teachers at each school who had received standardized training explained the research protocol and ethical safeguards to guardians for informed-consent collection. The research team provided unified training for participating homeroom teachers to clarify operational procedures and key implementation points for obtaining informed consent from minor participants. Participants could access the formal questionnaire page only after written guardian consent had been obtained and the adolescents themselves provided individual assent by clicking the “agree to participate” button on the questionnaire interface. Completion of the full questionnaire took approximately 15 min. Three attention-check items were embedded within the questionnaire to screen for invalid responses caused by careless or random answering. After questionnaire collection was completed, two independent researchers separately exported datasets for cross-verification. A total of 3, 323 questionnaires were collected. Invalid and incomplete responses were excluded based on performance on attention-check items and logical screening of questionnaire responses, yielding a final valid sample of 3, 009 participants, with an effective response rate of 90.55%.

### 2.2 Measuring tools

#### 2.2.1 Health literacy

Health literacy was measured using the 12-item short-form Health Literacy Survey questionnaire (HLS-SF12), which has been widely applied among Asian populations (38). The scale consists of 12 items and adopts a 4-point Likert-type rating scale, with response options ranging from 1 to 4. Participants responded based on their real-life capabilities regarding accessing, understanding, evaluating, and applying health-related information. The total scale score was computed by summing scores across all items; higher total scores indicate higher levels of Health literacy. The internal-consistency Cronbach’s *α* coefficient of this scale was 0.716 in the present study.

#### 2.2.2 Eating behaviors

Eating behaviors was measured using the short-form Eating behaviors scale sinicized by Ge et al. (39). The scale contains seven items corresponding to the following descriptions: having no regular mealtimes; feeling satisfied only after eating to full satiety; eating quickly; believing that one is more prone to weight gain than others; preferring high-fat foods; eating when other people around are eating; feeling satisfied only when purchasing more food than one’s actual needs. The scale adopts a 4-point Likert-type rating scale, where scores of 1 to 4 correspond to “strongly agree,” “disagree,” “agree,” and “strongly disagree,” respectively. Higher total scores indicate higher levels of healthy Eating behaviors among individuals. The Cronbach’s *α* coefficient for this scale was 0.922 in the present sample.

#### 2.2.3 School connectedness

School connectedness was measured using the School connectedness scale developed by Yu et al. (40). The scale consists of 10 items and comprehensively characterizes adolescents’ School connectedness across three dimensions: teacher support, peer support, and school belonging. The scale adopts a 5-point Likert-type rating scale with response options: 1 = strongly disagree, 2 = somewhat disagree, 3 = neutral, 4 = somewhat agree, 5 = strongly agree. Scores increase with higher levels of agreement. Higher dimension scores indicate stronger perceived teacher support, peer support, and school belonging among middle-school students. In the present study, the Cronbach’s *α* coefficients for the peer-support, school-belonging, and teacher-support dimensions were 0.850, 0.875, and 0.878, respectively; the overall Cronbach’s *α* coefficient for the full scale was 0.948.

#### 2.2.4 Physical activity participation

Physical activity participation was measured using the adolescent Physical activity participation scale originally developed by Zhi et al. and further validated by Qiu et al. (41, 42). This scale has been applied among Chinese populations and focuses on assessing the intrinsic motivational forces driving individuals to engage in physical exercise. The scale comprises six items that evaluate participants across six motivational dimensions: social-interaction enhancement, exercise-related pleasure experience, physical-health promotion, pursuit of motor skills, physical-appearance management, and stress release. The scale employs a 5-point Likert-type rating scale, and participants responded according to their actual degree of agreement with each item. The total score for Physical activity participation was computed by summing the scores of the six items. Higher total scores indicate stronger intrinsic motivation for physical-exercise engagement and higher intrinsic-motivation levels of Physical activity participation. The Cronbach’sαcoefficient of this scale was 0.703 in the present sample.

### 2.3 Statistical analysis

Descriptive statistics and correlation analyses were conducted using SPSS version 26.0, and the PROCESS macro was used to examine the mediation effects. Several procedural measures were implemented during questionnaire administration to minimize potential common method bias. First, respondents were assured of complete anonymity; no names or student ID information were collected, and participants were informed that only aggregated statistical results would be used for research purposes. Second, distinct introductory instructions were provided before each scale module to clarify the response frame for health literacy, school connectedness, eating behaviors, and physical-activity-participation. Third, three attention-check items were embedded in the questionnaire to identify and exclude careless or random responses. Item-order randomization was not implemented. Harman’s single-factor test extracted 13 factors with eigenvalues greater than 1. The first factor accounted for 30.193% of the total variance, below the 40.00% threshold, suggesting that no single factor dominated the variance structure and that severe common method bias was unlikely (43).

## 3 Research results

### 3.1 Descriptive statistics and correlation analysis

Means, standard deviations, and the Pearson correlation matrix for all variables are presented in Table 1. The results revealed significant positive pairwise correlations among Health literacy, School connectedness, Physical activity participation, and Eating behaviors. Significant correlations were observed among all key variables, providing empirical foundations for the subsequent test of the moderated-mediation model.

**Table 1:** Table of descriptive statistics and correlation analysis (*n* = 3,009).

Variable	M	SD	EB	SC	PAP	HL
EB	24.247	4.507	1	—	—	—
SC	38.985	7.825	0.065***	1	—	—
PAP	22.135	3.994	0.077***	0.624***	1	—
HL	39.617	4.544	0.235***	0.508***	0.448***	1

***p < 0.001.

### 3.2 Mediating effect analysis

To test the mediating role of School connectedness between Health literacy and Eating behaviors, hierarchical regression analysis combined with the Bootstrap resampling method (5,000 resamples)was adopted for mediation-effect testing, as illustrated in Figure 2. Mediation-path analyses revealed that the total effect of Health literacy on Eating behaviors was significant (*b* = 0.233, *p* < 0.001), indicating that Health literacy positively predicted healthy Eating behaviors among middle-school students. Hypothesis H1 was therefore supported. Further path analysis indicated that this effect operated through two distinct pathways. First, for the direct-effect pathway: after incorporating the mediator School connectedness, the direct predictive effect of Health literacy on Eating behaviors remained significant(*b* = 0.270, *p* < 0.001). Notably, the magnitude of the direct effect exceeded that of the total effect. Second, for the mediation-effect pathway: Health literacy significantly and positively predicted School connectedness (*b* = 0.875, *p* < 0.001), whereas School connectedness significantly and negatively predicted healthy Eating behaviors(*b* = −0.042, *p* < 0.001). The indirect effect mediated by School connectedness was negative, with an opposite sign relative to the total and direct effects, indicating a suppression effect. Although the indirect effect through School connectedness was statistically significant, its direction was negative and therefore opposite to the positive mediation hypothesized in H2. Accordingly, H2 was not supported in its hypothesized directional form. Instead, the results revealed an exploratory suppression pattern, whereby the indirect association through School connectedness operated in the opposite direction to the positive direct association between Health literacy and Eating behaviors.

**Figure 2:**
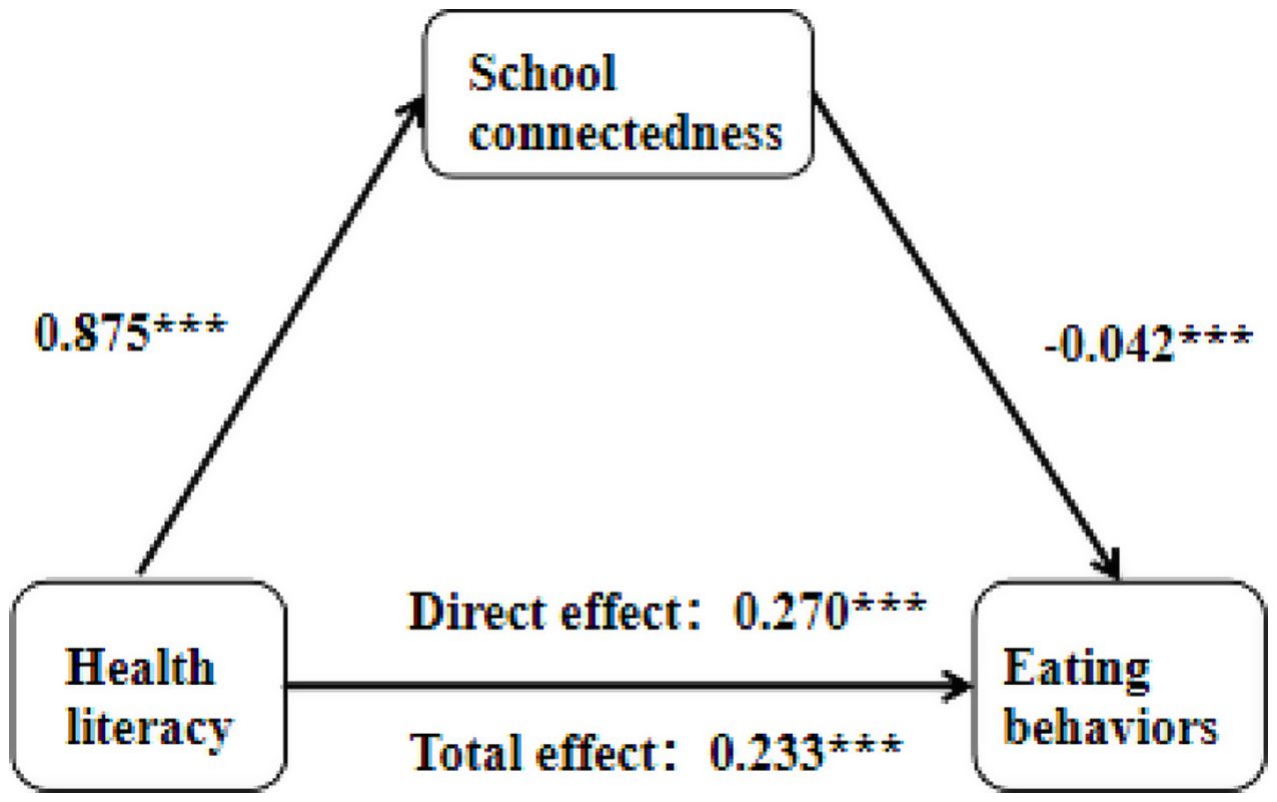
Verification of the mediating effect.

### 3.3 Moderated mediation effect test

A moderated mediation effect test was performed using PROCESS Macro Model 15 proposed by Hayes (2013). The Bootstrap resampling method was employed with 5,000 repetitions to estimate confidence intervals, and the results are presented in Tables 2–5.

**Table 2:** Regression analysis of the moderated mediation model.

Item	SC		EB	
	*b*	*t*	*b*	*t*
Gender	0.203	0.813	0.1	0.622
Age	−0.194	−1.321	0.016	0.167
HL	0.875	32.307	1.269	10.779
PAP	—	—	1.026	6.062
HL*PAP	—	—	−0.045	−8.57
SC	—	—	−0.471	−7.238
SC*PAP	—	—	0.019	6.63
*R* ^2^	0.259		0.083	
*F*	349.58***		38.691***	
Δ*R*^2^ (HL × PAP)	—	—	0.022	—
Δ*R*^2^ (SC × PAP)	—	—	0.013	—

*p < 0.05, **p < 0.01, ***p < 0.001.

**Table 3:** Conditional direct effect.

Level	Level value	Effect	LLCI	ULCI
−1SD	18.141	0.449	0.392	0.505
M	22.135	0.268	0.227	0.309
+1SD	26.129	0.088	0.028	0.147

**Table 4:** Conditional indirect effect.

Mediating variable	Level	Effect	BootSE	BootLLCI	BootULCI
SC	−1SD	−0.108	0.015	−0.142	−0.086
	M	−0.041	0.01	−0.061	−0.026
	+1SD	0.026	0.013	0.007	0.053

**Table 5:** The moderated mediation index.

Regulating variable	Mediating variable	Index	BootSE	BootLLCI	BootULCI
PAP	SC	0.017	0.002	0.013	0.022

To test the hypothesized moderated-mediation model (Hayes’ PROCESS Model 15), gender and age were included as control variables. All predictor variables were mean-centered prior to analysis. A total of 5,000 Bootstrap resamples were used to construct 95% bias-corrected confidence intervals. The overall regression coefficients for the model are presented in Table 2. The overall regression model predicting Eating behaviors was statistically significant (*R*^2^ = 0.083, *F* = 38.691, *p* < 0.001), indicating that the predictors included in the model jointly accounted for 8.3% of the variance in Eating behaviors. Beyond the regression coefficients, the Health literacy × Physical activity participation interaction uniquely accounted for an additional 2.2% of the variance in Eating behaviors (Δ*R*^2^ = 0.022), whereas the School connectedness × Physical activity participation interaction uniquely accounted for an additional 1.3% of the variance (Δ*R*^2^ = 0.013). These incremental contributions provide further statistical evidence for the moderating role of Physical activity participation in both the direct pathway from Health literacy to Eating behaviors and the second stage of the indirect pathway through School connectedness.

For the moderating effect on the direct path from Health literacy to Eating behaviors(Hypothesis H3a): The interaction term of Health literacy × Physical activity participation significantly predicted Eating behaviors, indicating that Physical activity participation significantly moderated the direct relationship between Health literacy and Eating behaviors. Simple-slope analysis was performed at three levels of Physical activity participation: low, mean, and high, and the results are presented in Table 3. At low levels of Physical activity participation, Health literacy exerted a relatively strong positive predictive effect on healthy Eating behaviors. At the mean level of Physical activity participation, this positive predictive effect decreased. At high levels of Physical activity participation, the positive predictive effect was further weakened yet remained statistically significant. Taken together, as the level of Physical activity participation increased, the direct positive predictive effect of Health literacy on healthy Eating behaviors gradually diminished. Hypothesis H3a was thus supported.

As shown in Table 5, the index of moderated-mediation was 0.017. The confidence interval did not contain zero, providing formal statistical evidence for the existence of the moderated-mediation effect. Taken together with the pattern of effect changes observed in Table 4, Hypothesis H3b was supported.

## 4 Discussion

### 4.1 The direct effect of health literacy on eating behaviors

The present study revealed a significant positive association between Health literacy and adolescents’ healthy Eating behaviors, supporting Hypothesis H1 and extending previous evidence linking literacy-related cognitive resources with dietary choices (11). Health literacy refers to individuals’ comprehensive ability to acquire, understand, and screen health-related information and translate such information into health-promoting behaviors, serving as a critical resource for facilitating the formation of healthy lifestyles (44). From the perspective of cognitive behavioral logic, adolescents with high Health literacy can effectively grasp nutritional knowledge, distinguish fragmented and misleading diet-related information in online environments, and establish scientific dietary cognition, thereby proactively adopting balanced dietary structures and developing regular and healthy eating habits. In contrast, individuals with low Health literacy are more susceptible to unscientific dietary misconceptions, struggle to transform health knowledge into daily dietary practices, and are more likely to develop unhealthy Eating behaviors. These findings are consistent with the view that cognitive resources provide an important basis for adolescents to evaluate health information and translate such information into behavioral choices (8, 45). More importantly, the theoretical contribution of the present study extends beyond confirming the positive association between Health literacy and healthy Eating behaviors. Rather than conceptualizing the cognition–behavior relationship as a simple and context-independent pathway, the present moderated-mediation framework shifts attention from whether Health literacy is associated with Eating behaviors to how and under what conditions this association varies. By simultaneously incorporating School connectedness as a school-contextual pathway and Physical activity participation as a behavioral boundary condition, the present study situates Health literacy within a broader cognition–context–behavior framework. From this perspective, the translation of Health literacy into healthy Eating behaviors may not be uniform across adolescents. Although Health literacy provides cognitive resources that facilitate healthy dietary decision-making, the extent to which these resources are reflected in Eating behaviors may depend on the social and behavioral contexts in which adolescents are embedded. In the present model, School connectedness represents a school-contextual pathway through which the association between Health literacy and Eating behaviors may operate, whereas Physical activity participation conditions both the direct Health literacy–Eating behaviors association and the latter segment of this indirect pathway. Thus, the cognition–behavior relationship is better understood as a conditional process in which individual cognitive resources operate in conjunction with school-contextual and behavioral factors rather than independently of them. This conditional perspective extends previous work by providing a more differentiated explanation of the potential cognition–behavior gap. Possessing adequate Health literacy does not necessarily imply that health-related knowledge and cognitive resources will be translated uniformly into corresponding Eating behaviors. Instead, the behavioral expression of these cognitive resources may vary according to adolescents’ School connectedness and Physical activity participation. Accordingly, the contribution of the present study lies not simply in identifying Health literacy as a positive correlate of healthy Eating behaviors, but in demonstrating that this association is embedded within a broader system involving individual cognition, school-contextual resources, and behavioral practices. This perspective also has implications for school-based health promotion. Although Health literacy education remains an important component of interventions designed to promote healthy Eating behaviors (46, 47), cognitive enhancement alone should not be assumed to translate uniformly into healthier dietary practices. School-based interventions may therefore benefit from considering the contextual and behavioral conditions under which Health literacy is translated into Eating behaviors, rather than treating Health literacy as an independently sufficient behavioral resource. Nevertheless, because the present study employed a cross-sectional design, these findings should be interpreted as evidence of conditional associations rather than established causal processes.

### 4.2 The mediating role of school connectedness

This study tested the mediating effect of School connectedness between Health literacy and Eating behaviors. Although a statistically significant indirect effect through School connectedness was identified, its direction was opposite to the positive mediation specified in H2. Therefore, H2 was not supported in its hypothesized directional form. Instead, the results revealed an exploratory suppression pattern. Specifically, the indirect effect through School connectedness was opposite in direction to the direct effect of Health literacy on Eating behaviors, indicating that School connectedness statistically suppressed the total association between Health literacy and Eating behaviors to some extent. Correlation analyses showed that Health literacy was significantly positively correlated with School connectedness, and Health literacy significantly positively predicted School connectedness. Students with higher Health literacy usually possess stronger information-screening and social-adaptation capacities. They are more receptive to school-delivered health guidance, institutional rules and values, and tend to develop school identity and a sense of belonging, which contributes to higher levels of School connectedness. Nevertheless, although the zero-order correlation between School connectedness and healthy Eating behaviors was statistically significant (*r* = 0.065, *p* < 0.001), the magnitude of this association was extremely small. Given the large sample size (*N* = 3, 009), statistical significance should not be interpreted as evidence of substantial practical importance. Therefore, the role of School connectedness in healthy Eating behaviors should be interpreted cautiously. Within the mediation model, School connectedness negatively predicted healthy Eating behaviors after accounting for Health literacy and the covariates, resulting in an indirect effect opposite in direction to the positive direct effect of Health literacy and thus a statistical suppression effect. However, given the very small zero-order association between School connectedness and healthy Eating behaviors, the practical magnitude of this suppression effect should not be overstated. Rather, the finding should be interpreted primarily as evidence of a statistically detectable pathway whose substantive relevance requires further verification. Importantly, the empirically observed suppression pattern should be distinguished from the possible mechanisms proposed to explain it. The present data demonstrate that the indirect association through School connectedness operated in the opposite direction to the positive direct association between Health literacy and Eating behaviors, but they do not establish why this statistical pattern emerged. Therefore, the following explanations should be regarded as non-mutually exclusive, theoretically plausible interpretations rather than mechanisms established by the present study. One plausible interpretation concerns peer dietary conformity. Previous evidence suggests that adolescents’ Eating behaviors may be associated with the dietary behaviors of their schoolmates and broader peer social networks (48). Similarly, peer-group dietary behavior has been associated with adolescents’ snack consumption (49). These findings raise the possibility that adolescents with stronger School connectedness may be more deeply embedded in school-based peer networks and consequently more exposed to prevailing peer dietary norms. Because such norms are not necessarily health-promoting, greater integration into particular peer groups could, under some circumstances, increase conformity to less healthy dietary practices and thereby weaken the translation of Health literacy into healthy Eating behaviors. However, peer dietary norms and dietary conformity were not directly measured in the present study. Therefore, peer dietary conformity should be regarded as one plausible interpretation of the observed suppression pattern rather than its principal or empirically established mechanism. However, peer dietary conformity is unlikely to be the only possible explanation. A second interpretation is that School connectedness may encompass heterogeneous school social experiences whose behavioral implications are not uniformly health-promoting. Formal health-promoting school environments can support healthier adolescent dietary and lifestyle behaviors (50), whereas informal social environments within the same school may involve more heterogeneous behavioral norms. Thus, the observed suppression pattern may partly reflect differences in the social contexts to which adolescents feel connected rather than peer dietary conformity specifically. A third possibility is that the suppression pattern may partly reflect unmeasured contextual or individual factors. Family dietary environments, food availability, socioeconomic conditions, peer-group composition, and other psychosocial characteristics may influence the associations among School connectedness and Eating behaviors. Because these factors were not comprehensively assessed in the present study, alternative explanations for the observed suppression pattern cannot be excluded. Taken together, the present findings do not support a simple characterization of School connectedness as either uniformly protective or inherently detrimental to healthy Eating behaviors. Rather, within the present mediation model, School connectedness functioned statistically as a counteracting pathway, partially offsetting the positive association between Health literacy and healthy Eating behaviors. This finding suggests that the association between School connectedness and health-related behaviors may be more context-dependent than a uniformly protective interpretation would imply. Nevertheless, given the extremely small zero-order association between School connectedness and healthy Eating behaviors and the absence of direct measures of the proposed contextual mechanisms, this finding should be regarded as exploratory and interpreted cautiously. The practical implications should therefore remain appropriately bounded. Shaping adolescents’ healthy Eating behaviors may require attention not only to individual Health literacy and School connectedness but also to the broader social and behavioral contexts in which these resources operate. However, the present findings do not establish that peer dietary culture causes the suppression pattern or that modifying peer dietary culture would necessarily eliminate it. Instead, school-based health promotion may benefit from considering the specific social and behavioral contexts in which School connectedness is embedded. It is noteworthy that although School connectedness constituted a statistically significant indirect pathway, the direct association between Health literacy and Eating behaviors remained significant. This suggests that School connectedness constitutes a relevant but non-exclusive indirect pathway for explaining the observed associations. Future research may adopt longitudinal tracking designs to further disentangle the temporal ordering among Health literacy, School connectedness, and Eating behaviors. Additional studies should directly assess peer dietary norms, dietary conformity, family dietary environments, and different school social contexts to determine which, if any, of these factors account for the observed suppression pattern. Such research would help distinguish among competing explanations rather than assuming that peer dietary subculture constitutes the primary mechanism underlying the suppression pattern.

### 4.3 The moderating role of physical activity participation

The present findings revealed that Physical activity participation exerted significant moderating effects on both pathways. As a critical individual lifestyle-related variable, Physical activity participation, measured by sports-participation motivation, served not only as a boundary condition for the effect of Health literacy on Eating behaviors but also moderated the latter segment of the indirect pathway through School connectedness. These findings further illustrate the boundary conditions underlying the association between Health literacy and Eating behaviors. Regarding Hypothesis H3a, conditional direct-effect analyses demonstrated that the magnitude of the direct effect of Health literacy on Eating behaviors varied markedly across different levels of Physical activity participation. At low levels of Physical activity participation, the direct effect of Health literacy on Eating behaviors was 0.449; at the mean level, this effect decreased to 0.268; and at high levels of Physical activity participation, it further decreased to 0.088. The confidence intervals excluded zero at all three levels, confirming the statistical significance of these conditional direct effects. Taken together, these findings indicate that as Physical activity participation increased, the direct positive association between Health literacy and healthy Eating behaviors was progressively attenuated. From the perspective of behavioral lifestyles, strong sports-participation motivation may promote adolescents’ subjective investment in school-based health-promoting physical-activity practices. Adolescents with high sports-participation motivation may be more likely to internalize healthy lifestyle principles through institutional school sport activities and develop coordinated exercise-diet lifestyle routines (51, 52). Under such circumstances, part of the behavioral support for healthy Eating behaviors may derive from habitual lifestyle patterns associated with sustained participation in school sports, thereby reducing the relative contribution of individual health-related cognition. In contrast, students with low sports-participation motivation may have fewer behavioral constraints and habitual supports derived from school sport engagement. Their Eating behaviors may therefore depend more strongly on cognitive-level Health literacy, resulting in a stronger association between Health literacy and Eating behaviors. Nevertheless, these behavioral explanations were not directly tested in the present study and should therefore be regarded as theoretically plausible interpretations of the observed moderation pattern. With respect to Hypothesis H3b, Physical activity participation moderated the latter segment of the mediating pathway, namely the relationship between School connectedness and Eating behaviors. Conditional indirect-effect analyses showed that the indirect effect through School connectedness was −0.108 at low Physical activity participation, −0.041 at the mean level, and shifted to 0.026 at high Physical activity participation. The confidence intervals did not contain zero across all three levels, and the index of moderated mediation was statistically significant. Hypothesis H3b was thus supported. These findings indicate that Physical activity participation altered both the direction and magnitude of the indirect effect through School connectedness. At low and mean levels of Physical activity participation, School connectedness exhibited a suppression pattern within the Health literacy–Eating behaviors pathway. At high levels of Physical activity participation, however, this suppression pattern was attenuated and statistically reversed, with the conditional indirect effect becoming weakly positive. Importantly, although the positive indirect effect at high Physical activity participation was statistically significant (indirect effect = 0.026, 95% CI [0.007, 0.053]), its magnitude was small and the lower confidence interval bound was close to zero. Therefore, this finding should not be interpreted as evidence of a substantial positive mediating effect of School connectedness. Rather, it primarily indicates a statistical attenuation and reversal of the suppression pattern at high levels of Physical activity participation. Accordingly, the educational and practical significance of this weak positive indirect effect remains uncertain and should be interpreted cautiously. One possible explanation for this conditional pattern concerns differences in the social contexts associated with sports participation. Students with relatively low sports-participation motivation may be more strongly embedded in routine class-based interactions, peer dining, and recreational activities. In such contexts, greater School connectedness could potentially increase exposure to and conformity with peer dietary norms, including unhealthy dietary practices, thereby contributing to the observed suppression pattern. By contrast, students with high sports-participation motivation may have greater opportunities to develop social connections through physical-education classes, extracurricular sports activities, and athletic clubs. These sport-oriented contexts may provide greater exposure to health-related information, including sports nutrition and balanced dietary practices, and may foster peer norms more compatible with healthy Eating behaviors. Under such circumstances, School connectedness may be less likely to operate as a counteracting pathway and may instead show a weak positive association within the indirect pathway. However, these explanations remain theoretically plausible interpretations rather than directly tested mechanisms, because peer dietary norms, sport-oriented social networks, and sports-related nutritional exposure were not directly measured in the present study. Future research should directly assess these contextual mechanisms before drawing firm conclusions regarding the processes underlying the observed moderated mediation pattern. Beyond its behavioral moderating role, the broader health relevance of physical activity should also be considered from a physiological perspective. Regular physical activity may confer benefits extending beyond behavioral regulation, including the maintenance of musculoskeletal function and potential neuroprotective effects. This perspective is particularly relevant given the emerging recognition of interactions between skeletal muscle and the central nervous system, with muscle atrophy and neurodegenerative processes sharing interconnected pathological mechanisms (53). Such physiological benefits may interact with dietary patterns and other health behaviors to influence long-term health outcomes. Although these physiological pathways were beyond the scope of the present behavioral study and were not directly assessed, integrating behavioral, musculoskeletal, and neuroprotective perspectives may provide a more comprehensive framework for understanding the broader health implications of Physical activity participation. Taken together, Physical activity participation appears to constitute a boundary condition that modifies the statistical pattern of the indirect pathway through School connectedness. However, given the small positive indirect effect observed at high levels of Physical activity participation, the present findings do not establish that increasing Physical activity participation would produce a practically meaningful beneficial effect of School connectedness on healthy Eating behaviors. Therefore, the current results should not be used to support specific educational interventions aimed at increasing Physical activity participation solely for the purpose of strengthening the School connectedness–healthy Eating behaviors pathway. Rather, they provide preliminary evidence that the role of School connectedness within the Health literacy–Eating behaviors pathway may vary across different levels of Physical activity participation. Whether this statistical reversal translates into meaningful educational or behavioral benefits requires further verification through longitudinal and intervention studies.

### 4.4 Limitations and future directions

Although this study used a moderated-mediation model to examine the mechanisms through which Health literacy shapes adolescents’ healthy Eating behaviors and yielded several important empirical findings, several limitations warrant consideration. First, this study employed a cross-sectional questionnaire-based design. All variables were measured using participants’ self-reports, allowing only the assessment of associations among variables rather than strict causal inference. Although the hypothesized model was grounded in the social-ecological theory, cross-sectional data cannot establish the temporal ordering of the variables. Future studies could employ cross-lagged or longitudinal designs with multiple waves of data collection to more rigorously evaluate the temporal and causal relationships proposed in the present moderated-mediation model. Second, all data were derived from participants’ subjective self-reports. Although the Harman single-factor test did not indicate substantial common-method bias, it is only a preliminary diagnostic and cannot rule out the influence of social-desirability bias. Participants may have overestimated their levels of Health literacy, physical-activity participation, and healthy Eating behaviors. Subjective perceptions of School connectedness may also be influenced by transient emotional states. Future research could adopt multi-source data collection strategies that integrate teacher ratings, peer nominations, and objective behavioral records, thereby reducing bias associated with exclusive reliance on self-report measures. Third, the present study identified a statistically significant suppression effect involving School connectedness. However, peer dietary norms and peer pressure were not directly assessed. Therefore, attributing this suppression effect to peer dietary subculture remains a *post hoc* interpretation that lacks direct empirical support. Moreover, the zero-order correlation between School connectedness and healthy Eating behaviors was small in magnitude. Although statistically significant, the association was weak in practical terms. Accordingly, the real-world implications of this mediating pathway should be interpreted cautiously and should not be overgeneralized. Future research should directly assess peer dietary norms and peer pressure to clarify the mechanisms underlying this suppression effect. Fourth, the present study used Hayes PROCESS Model 15 to test the moderating effects of Physical activity participation only on the direct pathway and the latter segment of the mediating pathway. Potential moderation of the X → M pathway was not examined. Future studies could extend the model to examine moderation along this pathway, thereby providing a more comprehensive understanding of the boundary conditions associated with Physical activity participation. In addition, this study examined only the overall level of physical-activity participation without distinguishing specific dimensions such as exercise type, exercise intensity, and activity-participation contexts. Peer-group characteristics and health beliefs may vary across exercise types. Future research should distinguish these dimensions to determine whether the observed model effects vary across exercise-related characteristics. Fifth, the present study provides an initial cross-sectional examination of the proposed relationships and did not include intervention experiments. Building on the observed associations and conditional patterns, future research could first use controlled school-based interventions to determine whether these statistical relationships translate into meaningful behavioral changes among adolescents with different levels of Physical activity participation. Such studies would provide stronger evidence for evaluating the potential practical value of integrating Health literacy education, School connectedness, and physical-activity-related strategies in school-based health promotion. Beyond human behavioral interventions, future translational studies may also adopt experimental animal models, including models of muscle atrophy, to systematically investigate the combined effects of dietary interventions and physical activity and to elucidate their potential causal and synergistic physiological mechanisms (54). Such experimental approaches may complement human studies by providing mechanistic evidence on diet–physical activity interactions at the physiological and molecular levels. However, because animal models cannot directly reproduce psychosocial constructs such as Health literacy or School connectedness, they should be regarded as complementary approaches for investigating biological mechanisms rather than as direct experimental tests of the present moderated-mediation model.

## 5 Conclusion

This study recruited middle-school students as participants. Based on Hayes PROCESS Model 15 for moderated-mediation analysis, the present research explored the influence of Health literacy on adolescents’ healthy Eating behaviors, examined the mediating role of School connectedness, and tested the moderating effects of Physical activity participation on the direct pathway and the latter segment of the mediating pathway. The main conclusions are summarized as follows: (1) Health literacy significantly and positively predicted adolescents’ healthy Eating behaviors. This direct effect remained stable after controlling for demographic variables including gender and age. Individual health-cognitive resources can exert direct influences on adolescents’ Eating behaviors practices. Hypothesis H1 was supported. (2) A statistically significant mediating effect of School connectedness was observed between Health literacy and healthy Eating behaviors. Nevertheless, rather than the hypothesized positive mediation, a suppression effect was identified. Health literacy positively predicted School connectedness, whereas School connectedness negatively predicted healthy Eating behaviors, leading to opposite signs for the indirect and direct effects. This finding indicates that School connectedness may function as a counteracting factor in the association between Health literacy and healthy Eating behaviors. Although Health literacy positively predicted School connectedness, higher School connectedness was associated with poorer healthy Eating behaviors within the mediation pathway, thereby partially offsetting the positive effect of Health literacy on healthy Eating behaviors. This finding indicates that School connectedness may function as a counteracting factor in the association between Health literacy and healthy Eating behaviors. Although Health literacy positively predicted School connectedness, higher School connectedness was associated with poorer healthy Eating behaviors within the mediation pathway, thereby partially offsetting the positive effect of Health literacy on healthy Eating behaviors. Accordingly, H2 was not supported in its hypothesized directional form. Instead, the significant negative indirect effect indicated an exploratory suppression pattern. (3) Physical activity participation significantly moderated the direct pathway from Health literacy to healthy Eating behaviors, supporting Hypothesis H3a. As the level of Physical activity participation increased, the direct predictive effect of Health literacy on healthy Eating behaviors gradually attenuated. The predictive power of Health literacy for healthy Eating behaviors was stronger among adolescents with low physical-activity participation. (4) Physical activity participation significantly moderated the latter segment of the mediating pathway, namely the relationship between School connectedness and healthy Eating behaviors, supporting Hypothesis H3b. The conditional indirect effect through School connectedness varied in both magnitude and direction across different levels of Physical activity participation. At low levels of Physical activity participation, a suppression pattern was observed, whereas at high levels of Physical activity participation, this suppression pattern was attenuated and statistically reversed, with the conditional indirect effect becoming weakly positive. Importantly, although this positive indirect effect was statistically significant (indirect effect = 0.026, 95% CI [0.007, 0.053]), its magnitude was small and the lower confidence interval bound was close to zero. Therefore, this finding primarily indicates a statistical attenuation and reversal of the suppression pattern rather than a substantial beneficial effect of School connectedness. The educational and practical significance of this weak positive indirect effect remains uncertain and should be interpreted cautiously. The index of moderated mediation was statistically significant, providing statistical support for the proposed moderated mediation model. Taken together, the present findings suggest that adolescent healthy Eating behaviors are associated with the interplay among individual Health literacy, School connectedness, and Physical activity participation. Improving Health literacy alone may not fully account for variations in healthy Eating behaviors, and the role of School connectedness within this pathway may differ across levels of Physical activity participation. These findings highlight the potential value of considering individual cognitive, school-contextual, and physical-activity-related factors jointly when developing school-based health-promotion strategies. However, given the small positive indirect effect observed at high levels of Physical activity participation and the cross-sectional nature of the present study, the findings do not provide sufficient evidence to recommend increasing Physical activity participation specifically to strengthen the beneficial effect of School connectedness on healthy Eating behaviors, nor do they yet justify stratified interventions based on Physical activity participation levels. Rather, the present results provide preliminary evidence for a context-dependent pattern that should be further examined in longitudinal and intervention studies before specific educational or practical recommendations are formulated.

## Data Availability

The original contributions presented in the study are included in the article/supplementary material, further inquiries can be directed to the corresponding author.
